# A case of herpes zoster uveitis with severe hyphema

**DOI:** 10.1186/1471-2415-14-74

**Published:** 2014-05-29

**Authors:** Yoko Okunuki, Junichi Sakai, Takeshi Kezuka, Hiroshi Goto

**Affiliations:** 1 Department of Ophthalmology, Tokyo Medical University, 6-7-1 Nishishinjuku, Shinjuku-ku, Tokyo 160-0023, Japan

**Keywords:** Herpes zoster uveitis, Zoster sine herpete, Hyphema, Anti-varicella zoster virus IgG, Enzyme immunoassay

## Abstract

**Background:**

Uveitis sometimes causes hyphema, but severe hyphema as a complication following herpes zoster uveitis has rarely been reported. We report a rare case of zoster sine herpete with unusually severe hyphema.

**Case presentation:**

A 41-year-old Japanese female developed hyphema filling almost one-half of the depth of the anterior chamber after a two-week history of unilateral anterior uveitis. Hyphema persisted for four weeks while sectorial iris atrophy became gradually apparent. Systemic prednisolone and valaciclovir resulted in prompt resolution of uveitis and hyphema. Serum anti-varicella zoster virus (VZV) IgG measured by enzyme immunoassay was 116 at presentation and decreased to 20.3 four month later. In addition, the antibody level in aqueous humor was almost 10-fold higher than that in serum examined 9 months after presentation. Because there was no skin lesion, this case was diagnosed as zoster sine herpete. The patient underwent cataract operation due to secondary cataract. The final visual acuity in decimal notation was 1.0, but complications such as severe iris atrophy, wide anterior synechiae, corneal opacity, and decrease in corneal endothelial cell count remained.

**Conclusion:**

Zoster sine herpete is an important differential diagnosis in a case of acute anterior uveitis with severe hyphema, although such cases are quite rare. Measurement of anti-VZV IgG levels by enzyme immunoassay in aqueous humor and serum would be useful in the diagnosis of VZV reactivation. Prompt diagnosis and administration of corticosteroids and anti-herpes virus medication may improve the outcome.

## Background

In this report, we present a case of acute anterior uveitis with unusually severe hyphema. Not many cases of uveitis develop hyphema. However, hyphema is known to develop in some anterior uveitides including herpetic uveitis, Fuchs heterochromic iridocyclitis, ankylosing spondylitis, Reiter’s syndrome, and chronic uveitis with rubeosis, although hyphema is mild in most cases [1,2].

Herpes zoster usually develops as reactivation of latent varicella zoster virus (VZV) infection after chicken pox. Typical herpes zoster involving the first branch of the trigeminal nerve with skin lesions is called herpes zoster ophthalmicus (HZO), whereas recurrence of herpes zoster without skin lesions is known as zoster sine herpete (ZSH). Herpes zoster uveitis may develop in both HZO and ZSH. The common ocular manifestations in herpes zoster uveitis are keratitis, iridocyclitis, and conjunctivitis [3]. Hyphema as a complication following herpes zoster uveitis has been reported in a few cases [4,5], and severe hyphema in only one case [5]. We report a rare case of ZSH with severe hyphema diagnosed by serum and aqueous humor levels of anti-VZV IgG.

## Case presentation

**Figure 1:**
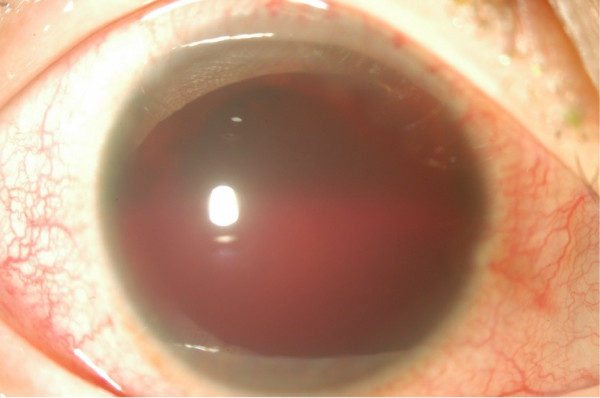
**An anterior photograph taken at presentation.** Prominent hyphema is visible, with apparent ciliary injection. Detail of the iris is not visible.

A 41-year-old Japanese female was referred to our department because of severe hyphema in the right eye for two days, and anterior uveitis that had persisted for two weeks. She had a history of chickenpox in early childhood, right HZO without ocular involvement at 11 years of age, and ovarian cyst. She had a headache and feeling of fatigue starting at the onset of ocular symptoms.At presentation, the best-corrected visual acuity (expressed in decimal scale) was counting finger at 30 cm OD and 1.0 OS. Intraocular pressure was 8 mmHg OD and 12 mmHg OS. Slit lamp examination of the right eye revealed ciliary injection and severe hyphema filling almost one-half of the depth of the anterior chamber (Figure 1). Due to the severe hyphema, there was no view of the fundus. However, no apparent abnormality was detected in B-mode echo examination. There was no rash on her face. She was receiving topical treatment with 0.1% betamethasone, 1% atropine, and anti-glaucoma agents, because intraocular pressure in the right eye was 30 mmHg when measured at the previous clinic before hyphema developed. Routine blood tests showed no abnormalities including blood cell counts, C-reactive protein, immunoglobulins (IgG, IgA, and IgM), and rheumatoid factor. Only anti-VZV IgG measured by enzyme immunoassay (EIA) (negative: < 2.0) was elevated to 116. Anti-herpes simplex virus IgG tested by EIA and tuberculin skin test (Mantoux test) were negative. Carotid ultrasound was performed to exclude the possibility that hyphema was caused by ocular ischemia, but there was no obstruction. There was no difference in blood pressure measured in two arms, which would exclude ocular ischemia caused by Takayasu disease. Since the presence of anterior inflammation was evident at presentation, subconjunctival injection of betamethasone (2 mg) was given and the topical medications prescribed by the former clinic were continued.

Two weeks after presentation, hyphema filling one-third of the anterior chamber persisted. Acetazolamide (500 mg/day) was started because intraocular pressure in the right eye increased to 28 mmHg and subconjunctival injection of betamethasone (2 mg) was given twice for persisting anterior inflammation. Four weeks after presentation, hyphema was approximately one-quarter of the depth. Visibility of anterior chamber was improved, and segmental iris atrophy that is one of the characteristic ocular manifestations of herpes zoster uveitis was visible. However, no facial skin lesion was observed. Detailed history taking revealed that she had hypersensitivity at the right forehead just before ocular symptoms appeared. Therefore, ZSH was suspected.

**Figure 2:**
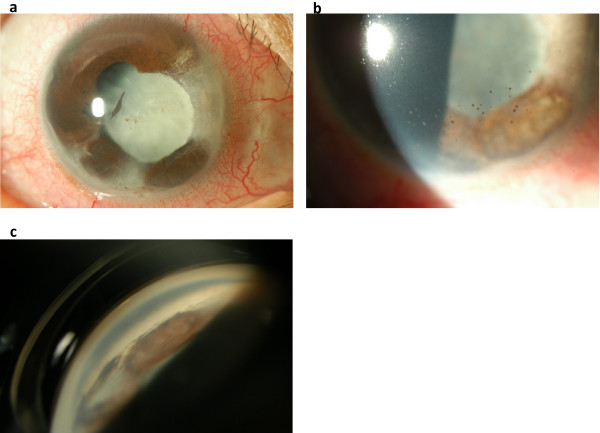
**Anterior and gonioscopic photographs taken three months after presentation, when inflammation and hyphema had almost resolved. (a)** Segmental iris atrophy and posterior synechiae from 12 to 8 o’clock position, and secondary cataract are apparent. Ciliary injection is better depicted than at presentation. **(b)** Pigmented keratic precipitates are found in the inferior cornea. **(c)** A gonioscopic photograph of inferonasal position. Broad anterior synechiae extends to posterior cornea.

Because anterior inflammation with ciliary injection and hyphema with fresh bleeding from atrophic area of the iris persisted, oral prednisolone (30 mg/day) and valaciclovir (3000 mg/day) were started. After starting these medications, ciliary injection improved and bleeding from the iris stopped. Then prednisolone and valaciclovir were gradually tapered. Oral medications were continued for 6 weeks and stopped, when ciliary injection became very mild and hyphema completely disappeared.At three months after presentation, visual acuity was 0.9 OD, and ocular pressure was 9 mmHg OD and 17 mmHg OS. Inflammation and hyphema were almost resolved (Figure 2a). Ophthalmoscopic examination revealed 1+ cell in the anterior chamber, pigmented keratic precipitates, segmental iris atrophy and posterior synechiae from 12 to 8 o’clock position, corneal opacity corresponding to the area of iris atrophy, and broad anterior synechiae extending to posterior cornea (Figure 2a-c). These observations were consistent with herpes zoster uveitis. Also, cataract developed but fundus seemed to be normal. Topical medication with 0.1% betamethasone was continued.

We then performed an anterior chamber paracentesis, since hyphema was resolved. Multiplex polymerase chain reaction (PCR) was conducted using the aqueous humor sample to detect viruses including VZV, herpes simplex virus, and cytomegalovirus. No viral DNA was detected. However, serial measurements of serum anti-VZV IgG showed a gradual decrease in level (Table 1). At 9 months after presentation, we measured anti-VZV IgG level (EIA) in aqueous humor and serum samples, and the levels were 269 and 20.3, respectively (Table 1). ZSH was thus confirmed.

**Table 1:** Serial anti-VZV IgG levels in serum and level in aqueous humor

	**VZV (IgG) EIA**	**Sample**
Oct 2011	116	Serum
Nov 2011	80	
Dec 2011	48.5	
Feb 2012	22.2	
Jul 2012	20.3	
	269	Aqueous humor

There was no recurrence of severe inflammation, but mild ciliary injection and a few cells in the anterior chamber persisted, and cataract gradually increased in severity. Twenty-two months after presentation, visual acuity was hand motion at 30 cm due to cataract. The patient underwent cataract operation. The surgery was uneventful. Corneal endothelial cell count was 989/mm^3^ before surgery, and remained almost the same after surgery. Visual acuity in the right eye improved to 1.0 after surgery. However, intraocular pressure fluctuated between 5 and 30 mmHg after surgery, and macular edema developed which was resolved by subtenon injection of triamcinolone.

## Discussion

We encountered a case of acute anterior uveitis with severe hyphema following ZSH in an immunocompetent woman. Several complications resulted, such as severe iris atrophy, decrease in corneal endothelial cell count, and secondary cataract necessitating operation.

A definitive diagnosis of ZSH can be made by detecting viral DNA in aqueous humor [6], but antibody examination is also useful in diagnosis [7]. In this case, PCR was performed three months after onset when inflammation had almost resolved, and the result was negative. We did not perform anterior chamber paracentesis while hyphema was present to avoid aggravating the hyphema. Delay of the procedure might be the reason that VZV-DNA was not detected by PCR. We next attempted to detect virus-specific antibody in the aqueous humor. Even at 9 months after onset, anti-VZV IgG (EIA) in the aqueous humor was approximately 10-fold higher than the serum level. Because an antibody ratio equal to or higher than 1.0 suggests local antibody production [7], VZV was the probable cause of anterior uveitis. A positive serum anti-VZV IgG test by EIA usually indicates past VZV infection. The EIA is more sensitive and specific than the complement fixation test for VZV [8]. In this case, it was impossible to examine paired (acute and convalescent) serum samples for IgG antibodies, because the ocular disease had persisted for more than two weeks when the patient presented. However, gradual decrease of serum anti-VZV IgG in 4 months (Table 1) after disease onset strongly suggests that uveitis was caused by VZV.

The diagnostic approach of measuring anti-VZV IgG (EIA) in the aqueous humor and serum is useful when detection of viral DNA in aqueous humor by PCR is negative, as in the present case. However, detection of viral DNA in aqueous humor should remain the gold standard for establishing a definitive diagnosis of ZSH [6]. On the other hand, the present diagnostic approach may be superior to conventional paried serum test, because it provides additional information of local production of specific IgG in ocular tissues. However, IgG antibodies do not reach high levels in the early phase of disease. Therefore, the sensitivity of this method would be low if peformed in the early phase of disease.

In most cases of uveitis with hyphema, the cause of bleeding is supposed to be rubeosis [1]. In herpes zoster uveitis, however, iris fluorescein angiography suggests occlusive vasculitis to be the main pathogenesis of the iris lesion causing bleeding [4,9]. The presence of rubeosis cannot be excluded in the present case, because iris fluorescein angiography was not performed, but marked iris atrophy observed after resolution of inflammation suggests the presence of severe inflammation that may cause obstruction of iris vessels.

In the present case, the ocular condition improved significantly after administration of systemic prednisolone and valaciclovir. Prednisolone was used for the anti-inflammatory effect and valaciclovir for suppression of the reactivated VZV. We cannot deny that more prompt administration of systemic medications might have reduced the complications such as secondary cataract, decrease in corneal endothelial cell count, iris atrophy, and corneal opacity.

## Conclusions

We propose that ZSH is an important differential diagnosis in a case of acute anterior uveitis with severe hyphema. Serial measurements of serum anti-VZV IgG (EIA) and simultaneous measurements of serum and aqueous humor anti-VZV IgG (EIA) led to a diagnosis for VZV reactivation. Especially in rare cases of severe anterior inflammation with marked hyphema, laboratory tests for a diagnosis of VZV activation and prompt systemic administration of corticosteroids and anti-herpes virus medication may improve the outcome.

### Consent

Written informed consent was obtained from the patient for publication of this case report and accompanying images. A copy of the written consent is available for review by the Editor of this journal.

## Abbreviations

EIA: Enzyme immunoassay; HZO: Herpes zoster ophthalmicus; PCR: Polymerase chain reaction; VZV: Varicella zoster virus; ZSH: Zoster sine herpete.

## Competing interests

The authors declare that they have no competing interests.

## Authors’ contribution

YO participated in treatment of the patient and analysis and interpretation of data, and drafted the manuscript. JS participated in diagnosis and treatment of the patient, designed the structure of the manuscript, and critically revised the manuscript for important intellectual content. TK participated in acquisition of data, and critically revised the manuscript for important intellectual content. HG participated in analysis and interpretation of data, and critically revised the manuscript for important intellectual content. All authors have given final approval of the version to be published. All authors agree to be accountable for all aspects of the work in ensuring that questions related to the accuracy or integrity of any part of the work are appropriately investigated and resolved. All authors read and approved the final manuscript.

## Pre-publication history

The pre-publication history for this paper can be accessed here:

http://www.biomedcentral.com/1471-2415/14/74/prepub
